# Improving the adherence of type 2 diabetes mellitus patients with pharmacy care: a systematic review of randomized controlled trials

**DOI:** 10.1186/1472-6823-14-53

**Published:** 2014-07-07

**Authors:** Sunya-Lee Antoine, Dawid Pieper, Tim Mathes, Michaela Eikermann

**Affiliations:** 1 Institute for Research in Operative Medicine, Faculty of Health - School of Medicine, Witten/Herdecke University, Ostmerheimer Str. 200, Building 38, D- 51109 Cologne, Germany

**Keywords:** Adherence, Pharmacist intervention, Type 2 diabetes mellitus, Systematic review

## Abstract

**Background:**

Oral medication for patients with type 2 diabetes mellitus plays an important role in diabetes care and is associated with a high level self-care behavior and self-management. However, poor adherence to diabetes treatment is common which causes severe health complications and increased mortality. Barriers to adherence may consist of complex treatment regimens often along with long-term multi-therapies, side effects due to the medication as well as insufficient, incomprehensible or confusing information or instructions provided by the health care provider. Multidisciplinary approaches can support adherence success and can enable a more effective management of diabetes care. One approach in diabetes care can be the involvement of a pharmacist. The aim was to analyze the effectiveness of adherence-enhancing pharmacist interventions for oral medication in type 2 diabetes mellitus.

**Methods:**

A systematic review of randomized controlled trials. The study quality was assessed with the Cochrane risk of bias tool.

**Results:**

Of 491 hits, six publications were included. Two studies mainly examining educational interventions showed a significant improvement in adherence. Moreover, the quality of the included studies was deficient.

**Conclusion:**

Although pharmacist interventions might potentially improve adherence to type 2 diabetes mellitus medication, high-quality studies are needed to assess effectiveness.

## Background

Oral medication for patients with type 2 diabetes mellitus plays an important role in diabetes care and is associated with a high level self-care behavior and self-management [1]. However, poor adherence to diabetes treatment is common which causes severe health complications and increased mortality [2-4]. This is reflected for instance by an increase in the risk of cardiovascular diseases, neuropathy, retinopathy, nephropathy and hospitalization rates [3,5,6].

Barriers to adherence may consist of complex treatment regimens often along with long-term multi-therapies, side effects due to the medication as well as insufficient, incomprehensible or confusing information or instructions provided by the health care provider. Further barriers challenging adherence could also be related to socioeconomic issues, memory impairment, psychological well-being and personal beliefs [5,7,8].

Multidisciplinary approaches can support adherence success and can enable a more effective management of diabetes care. Several models for diabetes care have been developed and evaluated [9]. One approach in diabetes care can be the involvement of a pharmacist, especially since the role of a pharmacist has changed in the last decades. As the training of pharmacists and their responsibilities include more than just the manufacturing and administration of medicinal products, incorporating pharmacists in the direct care of diabetic patients could contribute to helping patients reach optimal adherence [10-13]. The responsibilities of pharmacists involve for example the long-term supervision, patient education activities, the consideration of medication-related issues (e.g. drug interactions) and of patient needs as well as the optimization of the medicinal treatment and adherence. Studies have shown that pharmacist interventions positively influence health outcomes and patient satisfaction, which are crucial indicators for quality of health care and a key factor for medication adherence [11].

A previous systematic review examined the effects of pharmacist interventions that improve adherence to oral antidiabetic medications for type 2 diabetes mellitus showing a positive effect on adherence [14]. However, even though a search for educational, behavioral, affective or provider-targeted strategies is described the provided search strategy is restricted to certain search terms which might lead to a non-identification of relevant publications. Further, the identified studies included in the review are merely described with respect to the study characteristics, types of interventions and study results, but, they are not systematically assessed for quality which impedes the extensive and concluding appraisal of the respective interventions. Moreover, the review included cohort studies in addition to randomized controlled trials aiming to provide exhaustive and generalizable results. Nevertheless, the consideration of non-randomized trials does not appear to augment the value of the review with respect to further outcome measures or longer follow-ups. Therefore, it was sought to perform a systematic review on randomized controlled trials analyzing the effectiveness of adherence-enhancing interventions involving pharmacists for oral medication in type 2 diabetes mellitus.

## Methods

### Literature search and selection criteria

A systematic search for relevant publications was conducted in bibliographic databases (Medline via EMBASE, EMBASE via EMBASE, CENTRAL via Cochrane Library) in March, 2013. A search strategy for each database was developed using medical subject headings and key words for adherence, pharmacist interventions and type 2 diabetes mellitus. The full search strategies are provided in Additional file 1. Randomized controlled/cluster-randomized controlled trials as full-text publications investigating pharmacist interventions in which a pharmacist is involved in the provision of the intervention to improve adherence, defined as the degree to which a patient follows the medical prescription in terms of interval and dose of a dosing regimen [15], to oral medication in type 2 diabetes mellitus were eligible for inclusion. If the type 2 diabetes mellitus medication could not be clearly classified as oral medication (e.g. metformin, alpha-glucosidase inhibitors, thiazolidinediones) the study was excluded. Moreover, the examined population had to consist of adult patients (≥18 years) and adherence to the oral medication in type 2 diabetes mellitus had to be measured. No limitation regarding the language or publication year of the studies was made.

### Study selection

Two independent reviewers screened the titles and abstracts of the identified publications according to the pre-defined criteria. After obtaining the full-texts of the potentially relevant publications two independent reviewers screened them and determined their eligibility for further analysis. If discrepancies regarding study inclusion could not be solved by discussion a third reviewer was involved.

### Data extraction

The results and study characteristics of each included study were then extracted and a second reviewer checked for accuracy and completeness. For this, standardized tables were used. These contained information on the first author, the publication year, the study type, the country and setting the trial took place, the study population size, age and sex as well as the content and length of the intervention and control intervention, the definition of adherence, the adherence measures and the adherence rate at baseline and last follow-up.

### Risk of bias

The risk of bias in the included studies was assessed by two independent reviewers according to pre-defined criteria based on the Cochrane risk of bias tool [16]. However, the criteria related to blinding of participants and personnel were not applicable. In adherence-enhancing interventions participants and the personnel delivering the intervention cannot be blinded due to the nature of the interventions. Thus, the respective criteria were not considered. Consequently, the criteria implemented to assess the methodological quality of the included studies consisted of questions related to the random sequence generation, the allocation concealment, blinding of outcome assessment, the analysis according to intention-to-treat, selective reporting and other sources of bias. If discrepancies regarding quality assessment could not be solved by discussion a third reviewer was involved. It was decided to rate each risk of bias item only as “yes” and “no” and not as “unclear” as recent research suggests that rating as “unclear” “becomes the default for the risk of bias tool assessments (RoB) regarding reliability” [17]. Moreover, it could be shown that a “significant difference in effect sizes […} between studies with a high or unclear risk of bias and those with a low risk of bias” exists [18].

## Results

**Figure 1:**
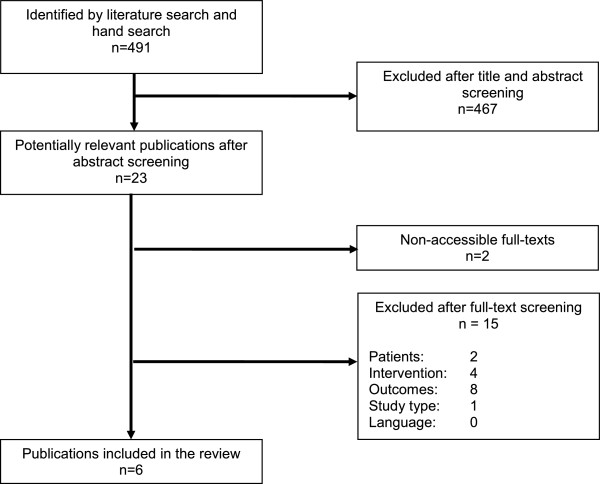
Flow diagram of study selection.

The literature search yielded a total of 491 articles (Figure 1). After screening titles and abstracts, 23 publications were considered as potentially relevant for further screening [9,19-40]. Of these, two publications were not obtainable and hence excluded. Eight studies were excluded mainly due to the missing measurement of adherence. Four studies did not have an intervention in which a pharmacist was actively involved in the provision of adherence-enhancing strategies for oral type 2 diabetes mellitus medication. In one study the examined study population were not adults (≥18 years), in one study the type 2 diabetes mellitus medication was not an oral medication and one study was not a randomized controlled trial. In total, six publications met the selection criteria and were included for further analysis [9,19-23].

The included studies showed qualitative deficits in terms of risk of bias (Table 1). In most studies the allocation sequence was not sufficiently generated (n = 4) as well as concealed (n = 5), potentially causing a selection bias. Further, the blinding of outcome assessment was not reported in five of the included studies and an analysis according to intention-to-treat was described in half of the included studies. In two studies a potential risk for selective reporting and other sources of bias could be identified. These are further depicted in the following for each study when describing the results.

**Table 1:** Risk of bias of included trials

**Study**	**Adepu (2010)**[19]	**Grant (2003)**[20]	**Mehuys (2011)**[9]	**Obreli-Neto (2011)**[21]	**Odegard (2005)**[22]	**Phumipamorn (2008)**[23]
**Random generation of allocation sequence**	-	-	+	-	-	+
**Allocation concealment**	-	-	+	-	-	-
**Blinding of outcome assessment**	-	-	-	+	-	-
**Analysis according to intention-to-treat**	-	-	+	+	+	-
**Selective reporting**	-	-	+	-	+	-
**Other sources of bias**	+	-	+	-	-	-

**+** fulfilled, **-** not fulfilled.

Among the included randomized controlled trials one study was a cluster-randomized controlled trial in which the participating pharmacists were randomly assigned to the intervention or control group [9]. The investigated interventions consisted of educational interventions supporting the correct medication use as well as reminders and counseling interventions, provided by pharmacists in cooperation with the treating physician in different settings and countries, e.g. outpatient health care facilities, pharmacies and hospital settings (Table 2).

**Table 2:** Study results

**Author, Year**	**Adepu (2010)**[19]	**Grant (2003)**[20]	**Mehuys (2011)**[9]	**Obreli-Neto (2011)**[21]	**Odegard (2005)**[22]	**Phumipamorn (2008)**[23]
**Study type**	RCT	RCT	Cluster-RCT	RCT	RCT	RCT
**Country/Setting**	South India/Medicine Outpatient Department Tertiary care teaching hospital	USA/Academically affiliated community health center	Belgium/Community pharmacies	Brazil/Public Primary Health Care Unit	USA/University of Washington Medicine Clinics	Thailand/Community Hospital
**Population (IG/CG)**	n = 227	62/58	153/135	97/97	39/27	63/67
**Age (IG/CG)**	>57	64 ± 12/69 ± 10	62 (45-79)/63 (40-84)	65 ± 5.8/65 ± 5.7	52 ± 11.6/52 ± 10.4	52 ± 11.15/56 ± 13.67
**Sex (IG/CG)**	49% female	55%/69% female	54%/51% male	63%/62% female	48%/48% female	92%/76% female
**Intervention**	Education (Baseline, every 30 days for a period of 3 months)	Educational telephone interview + social services/nutrition consultation (Baseline, 3 months)	Education & Reminders about annual eye and foot examinations (Baseline, at each prescription refill visit for a period of 6 months)	Usual care + individual follow-up attendances & educative group activities (every 6 months for period of 36 months)	Diabetes care plan DCP) & Weekly in person/telephone meetings & monthly follow-up phone calls (6-month intervention, follow-up at month 6 and 12)	Usual care & 4 scheduled meetings with a pharmacist (every 2 months) & education
**Control**	Education (month 3)	Educational telephone interview	Usual care	Usual care	Usual care	Usual care
**Definition adherence**	Average change in adherence (0-4 scale)	Amount of missed medication in the last 7 days (change from baseline)	Proportion of doses taken (%)	Proportion of doses taken (%)	Proportion of missed doses (%)	Proportion of doses taken (%)
**Adherence measure**	Self-reported adherence	Self-reported adherence	Prescription refill rate & self-reported adherence	Self-reported adherence & periodicity of prescription pickup	Self-reported adherence	Pill count
**Adherence (IG/CG)**						
Baseline	0.73/1.11	6.7/6.9	NR	51/49	56/35	82/87
				53/53		
Final (%)	0.88/0.67	0.1/0.1 (change from baseline)	99.7/94.7 (prescription refill rate)	84/44 (self-reported adherence)	IG < CG	89/85
	p = NR	p = 0.8	p = NR	84/43 (periodicity of prescription pickup)	p = 0.003	p = 0.004
			61/62 (self-reported adherence)	p = NR		
			p = NR			

NR = Not reported, IG = intervention group, CG = control group.

In five studies [9,19-21,23] educational interventions (e.g. by telephone or as group activities) addressing topics such as disease, medication, diet, and lifestyle modification were evaluated. In three of these studies educational interventions were provided in addition to social services and nutrition consultation as well as reminders about annual eye and foot examinations, individual follow-up attendances, scheduled meetings with a pharmacist and/or usual care [9,20,21]. One study investigated the implementation of a Diabetes Care Plan in addition to weekly in-person or telephone meetings and monthly follow-up phone calls [22]. Most interventions were compared with usual care [9,21-23] whereas Adepu et al. and Grant et al. used a cut-down provision of educational interventions as the comparator [19,20]. The duration and intervals of the interventions varied across all studies (from three to 36 months and from every 30 days to every 6 months).

In four of the studies adherence was defined as the proportion of medication taken [9,21-23], in one study the average change in adherence and in one study the average change of the amount of missed medication in the last 7 days were measured [19,20]. Self-reported adherence was used in almost all studies to measure adherence [9,19-21,23]. The prescription refill rate [9], the periodicity of prescription pickups [21] were used in addition in two studies and pill count was used to measure adherence by Phumipamorn [23]. Detailed information on country, setting, population size as well as age and sex can be found in Table 2.

In all studies a tendency of the pharmacist intervention for improving adherence to type 2 diabetes mellitus medication was reported, however, a statistically significant effect was shown in only the studies by Odegard et al. (p = 0.003) and Phumipamorn et al. (p = 0.004) (Table 2) [9,19-23]. Odegard et al. investigated a Diabetes Care Plan combined with weekly in-person and/or telephone meetings and monthly follow-up telephone calls provided by pharmacists against usual care [22]. They found that the adherence in the intervention group was significantly higher than in the control group, however, no adherence rates were reported [22]. Phumipamorn et al. demonstrated that the provision of scheduled meetings with a pharmacist alongside with the physician’s appointment increased adherence significantly compared with usual care. Adepu et al. showed that education provided regularly (at baseline and every 30 days for a duration of three months) compared to just one education session (at month three) tended to improve adherence [19]. However, the adherence between intervention and control group differed considerably at baseline, in spite of the fact that the study was a randomized controlled trial. It was not reported whether the differences at baseline were adjusted for in their analysis. The educational telephone interviews in addition to social services and nutrition consultations provided and arranged by a pharmacist examined by Grant et al. reduced the amount of missed medication. Though, the control group receiving only the educational telephone interviews showed also an almost perfect adherence in both groups at final measurement [20]. In the cluster-randomized trial by Mehuys et al. higher adherence to antidiabetic medication in the intervention group receiving education and reminders about annual eye and foot examinations compared to usual care in the control group was reported. But the baseline adherence of the intervention and control group was not reported. In addition, the possibility of cluster effects and the significance of the study results were not described. Further, in this study all patients with an adherence of >100% were excluded from the analysis [9]. Usual care complemented by a pharmaceutical care intervention consisting of individual follow-up attendances and educative group activities was compared to usual care by Obreli-Neto et al. and appeared to improve adherence, but no statistically significant effect was described [21,23].

## Discussion

The performed systematic review searched and analyzed randomized controlled trials on pharmacist interventions for patients taking oral type 2 diabetes medication with respect to adherence. In all six included studies the effect direction was in favor of the pharmacist interventions on improving adherence to antidiabetic medication. Overall, of the six included studies two studies showed a statistically significant effect of a Diabetes Care Plan combined with weekly in-person and/or telephone meetings and monthly follow-up telephone calls provided by pharmacists and of a pharmaceutical care intervention consisting of the provision of scheduled meetings with a pharmacist alongside with the physician’s appointment compared with usual care [22,23].

However, the included studies contain in parts heterogeneous interventions as well as different methods to define, to operationalize and to measure adherence only allowing for a comparison to a limited extent. In five studies [9,19-21,23] educational interventions (e.g. by telephone or as group activities) addressing topics such as disease, medication, diet, and lifestyle modification were evaluated. In three of these studies educational interventions were provided in addition to social services and nutrition consultation as well as reminders about annual eye and foot examinations, individual follow-up attendances, scheduled meetings with a pharmacist and/or usual care [9,20,21]. Most interventions were compared with usual care [9,21-23] whereas Adepu et al. and Grant et al. used a cut-down provision of educational interventions as the comparator [19,20]. In addition, as mentioned, self-reported adherence as well as the prescription refill rate, the periodicity of prescription pickups and pill count were mainly implemented as the adherence measure in the included studies. Although these represent adherence measures commonly implemented, they might be subjected to overestimation of adherence [41,42].

Furthermore, besides changes in adherence rates all included studies measured in addition relevant clinical outcomes such as blood glucose and blood pressure values as their reduction and maintenance are key aims in diabetes care to prevent possible complications and to achieve health gains in diabetic patients [34]. Statistically significant changes in blood pressure and blood glucose levels were found in favor of the intervention groups receiving pharmaceutical care in the majority of the studies [9,19,21-23]. Other relevant outcomes such as knowledge and self-management as factors affecting adherence were also assessed. The involvement of a pharmacist contributed to an improvement of knowledge and self-care activities in three studies [9,19,23]. However, different instruments were used for the assessment and knowledge as well as self-management values at the baseline and final assessment varied within and between the study groups among the studies. Moreover, the sample size was not adequately calculated in almost all of the studies or the sample size calculation was not reported [9,19-22].

A possible limitation is that pharmacists might individually differ in the way they provide their adherence-enhancing intervention. Additionally, they might show differences in identifying individual medication-related issues and patient needs, the intensity of the pharmacist-patient contact as well as in education and communication skills causing variances in outcomes. This issue has also been noted in other related publications [43,44]. Moreover, an aspect to be considered is the fact that pharmacists in their respective health care systems, in which the studies were conducted, are differently integrated in the health care provision [45]. For instance, in some health care systems pharmacist care might be more established and integrated as an organized element in the management of diseases as in other health care systems. Aspects such as education, professionalization, recognition and reimbursement just to mention some are essential influencing factors related to the differences in pharmacy care [46,47]. The differences in the role of pharmacists in different countries contribute to the difficulty in comparing the different pharmacist interventions. Hence, making a generalized conclusion remains difficult, especially against the background that the analyzed randomized controlled trials are conducted in various different countries with varying living circumstances and cultural backgrounds.

We could not judge in how far the results of our quality assessment are in line with the quality assessment by Omran et al. as their results are not depicted in detail. In addition to the randomized controlled trials also identified by Omran et al. our review identified three further relevant randomized controlled trials. Two studies by Al Mazroui et al. and Skaer et al. [34,39] which were included in the review by Omran et al. were not included in our review as they either did not fulfill our inclusion criteria or were not accessible.

The influence of pharmacist interventions in increasing adherence has been demonstrated in several publications, showing that the results of our review are in line with those of other publications, however, in how far health outcomes, quality of life or cost-effectiveness are improved is ambiguous [10,11,14,44,48]. Thus, further studies of high quality are needed to assess significant effectiveness of adherence-enhancing pharmacist interventions care, especially against the background that the study quality of the included trials in this review are deficient [14,49,50].

## Conclusion

Our review shows the existing evidence on the effectiveness of pharmacist interventions to enhance adherence in patients suffering type 2 diabetes mellitus. The outcomes of the analyzed studies indicate that pharmacists could have an influential and important role in the respective health care system to improve adherence in patients taking oral type 2 diabetes mellitus medication. However, the heterogeneity of study populations interventions, adherence measures and outcomes in the included studies prevents a comparison as well as a generalization. Besides, our review points out the lack of randomized controlled trials of pharmacist interventions in oral type 2 diabetes mellitus medication. Nevertheless, pharmacists should be further considered as an integral component in the health care provision for type 2 diabetes mellitus care, especially in terms of helping patients to reduce non-adherence and hence to improve health outcomes in patients taking oral type 2 diabetes mellitus medication. Future randomized controlled trials should be sought for to provide comparable results of outcomes.

## Competing interests

The authors declare that they have no competing interests.

## Authors’ contributions

SLA: research question development, search strategy development, study selection, data analysis, interpretation of results, preparation of the manuscript. DP: research question development, search strategy development, study selection, interpretation of results, review of the manuscript. TM: research question development, search strategy development, study selection, data analysis, interpretation of results, review of the manuscript. ME: research question development, search strategy development, review of the manuscript. All authors read and approved the final manuscript.

## Pre-publication history

The pre-publication history for this paper can be accessed here:

http://www.biomedcentral.com/1472-6823/14/53/prepub

## Supplementary Material

Additional file 1 Search strategy. Click here for file
