# Supplementary material for: Improving the adherence of type 2 diabetes mellitus patients with pharmacy care: a systematic review of randomized controlled trials
Source: BMC Endocr Disord. 2014 Jul 7;14:53. doi: 10.1186/1472-6823-14-53 (PMC4105396; doi:10.1186/1472-6823-14-53)
Supplement: Additional file 1 — Search strategy. [file 1472-6823-14-53-S1.docx]

Additional file 1: Search strategy

| **Database** | **Search strategy** |
| --- | --- |
| ***EMBASE (via EMBASE includingMEDLINE records)*** | ('pharmacist'/exp OR pharmacist*:ab,ti OR 'pharmacy':ab,ti OR 'pharmacy'/exp AND ('intervention':ab,ti OR 'participation':ab,ti OR 'care':ab,ti))  OR ('pharmaceutical care'/exp OR 'pharmaceutical care':ab,ti OR ('pharmaceutical':ab,ti OR 'drug'/exp OR 'drug':ab,ti OR 'medication':ab,ti OR medicine*:ab,ti AND 'monitoring':ab,ti))  OR ('drug monitoring':ab,ti OR 'drug monitoring'/exp)  OR ('consultant':ab,ti OR 'community'/exp OR 'community':ab,ti AND ('pharmacist'/exp OR 'pharmacist':ab,ti OR 'pharmacists':ab,ti OR 'pharmacy':ab,ti OR 'pharmacy'/exp OR 'pharmacies':ab,ti))  AND (adherence:ab,ti OR adherent:ab,ti OR adhere:ab,ti OR nonadherence:ab,ti OR nonadherent:ab,ti OR compliance:ab,ti OR 'patient compliance'/exp OR 'patient compliance' OR compliant:ab,ti OR comply:ab,ti OR noncompliance:ab,ti OR noncompliant:ab,ti AND ('drug therapy'/exp OR 'drug therapy' OR pharmacotherap*:ab,ti OR drug*:ab,ti OR pill*:ab,ti OR medicament*:ab,ti OR medication*:ab,ti OR medicin*:ab,ti OR pharmaceutical*:ab,ti OR tablet*:ab,ti OR treatment*:ab,ti OR intervention*:ab,ti) NOT ('children'/exp OR 'children') AND ('randomized controlled trial':it OR 'controlled clinical trial':it OR randomized:ab,ti OR randomised:ab,ti OR placebo:ab,ti OR 'clinical trial (topic)'/exp OR 'clinical trial (topic)' OR randomly:ab,ti OR trial:ab,ti) NOT ('animals'/exp OR animals NOT ('humans'/exp OR humans)) AND (english:la OR german:la) AND ('article'/it OR 'article in press'/it OR 'review'/it))  AND 'diabetes mellitus'/exp OR 'diabetes mellitus' OR 'diabetes mellitus':ab,ti OR 'non insulin dependent diabetes mellitus'/exp OR 'non insulin dependent diabetes mellitus' OR ('non insulin dependent diabetes mellitus':ab,ti OR 'type 2 diabetes mellitus':ab,ti OR 't2dm':ab,ti OR 'hypergyclemia' OR 'hyperglyclemia':ab,ti OR 'metabolic disorder'/exp OR 'metabolic disorder':ab,ti) |
| ***Cochrane Library (Central via Cochrane Library)*** | ((pharmacist [mesh] OR pharmacy [mesh] OR pharmacist: ti,ab OR pharmacist*: ti,ab (pharmacies OR pharmacy):ti,ab AND ((intervention OR participation OR care):ti,ab OR (consultant OR community):ti,ab) OR Community Pharmacy Services [mesh] OR (((Pharmaceutical OR drug OR medicin* OR medication):ti,ab AND monitoring:ti,ab) OR Drug Monitoring [mesh])  AND (Adherence OR adherent OR adhere OR nonadherence OR nonadherent OR Compliance OR compliant OR comply OR noncompliance OR noncompliant):ti,ab AND (Pharmacotherap* OR Drug* OR pill* OR medicament* OR medication* OR medicin* OR pharmaceutical* OR tablet* OR treatment* OR intervention*):ti,ab  AND (Diabetes Mellitus [mesh] OR Hyperglycemia [mesh] OR Glucose Metabolism Disorders [mesh] OR Diabetes Mellitus, Type 2 [mesh] OR diabetes mellitus:ti,ab OR non insulin dependent diabetes mellitus:ti,ab OR type 2 diabetes mellitus:ti,ab OR T2DM:ti,ab OR hyperglyclemia:ti,ab OR metabolic disorder:ti,ab) |
